# Author Correction: Direct observation of excitonic instability in Ta_2_NiSe_5_

**DOI:** 10.1038/s41467-021-23476-3

**Published:** 2021-05-14

**Authors:** Kwangrae Kim, Hoon Kim, Jonghwan Kim, Changil Kwon, Jun Sung Kim, B. J. Kim

**Affiliations:** 1 Department of Physics, Pohang University of Science and Technology, Pohang, South Korea; 2 Center for Artificial Low Dimensional Electronic Systems, Institute for Basic Science (IBS), Pohang, South Korea; 3 Department of Materials Science and Engineering, Pohang University of Science and Technology, Pohang, Republic of Korea

**Keywords:** Electronic properties and materials, Phase transitions and critical phenomena

Correction to: *Nature Communications* 10.1038/s41467-021-22133-z, published online 30 March 2021.

The original version of this Article contained an error in Fig. 1, in which the data was not corrected for the Bose factor.

The correct version is:
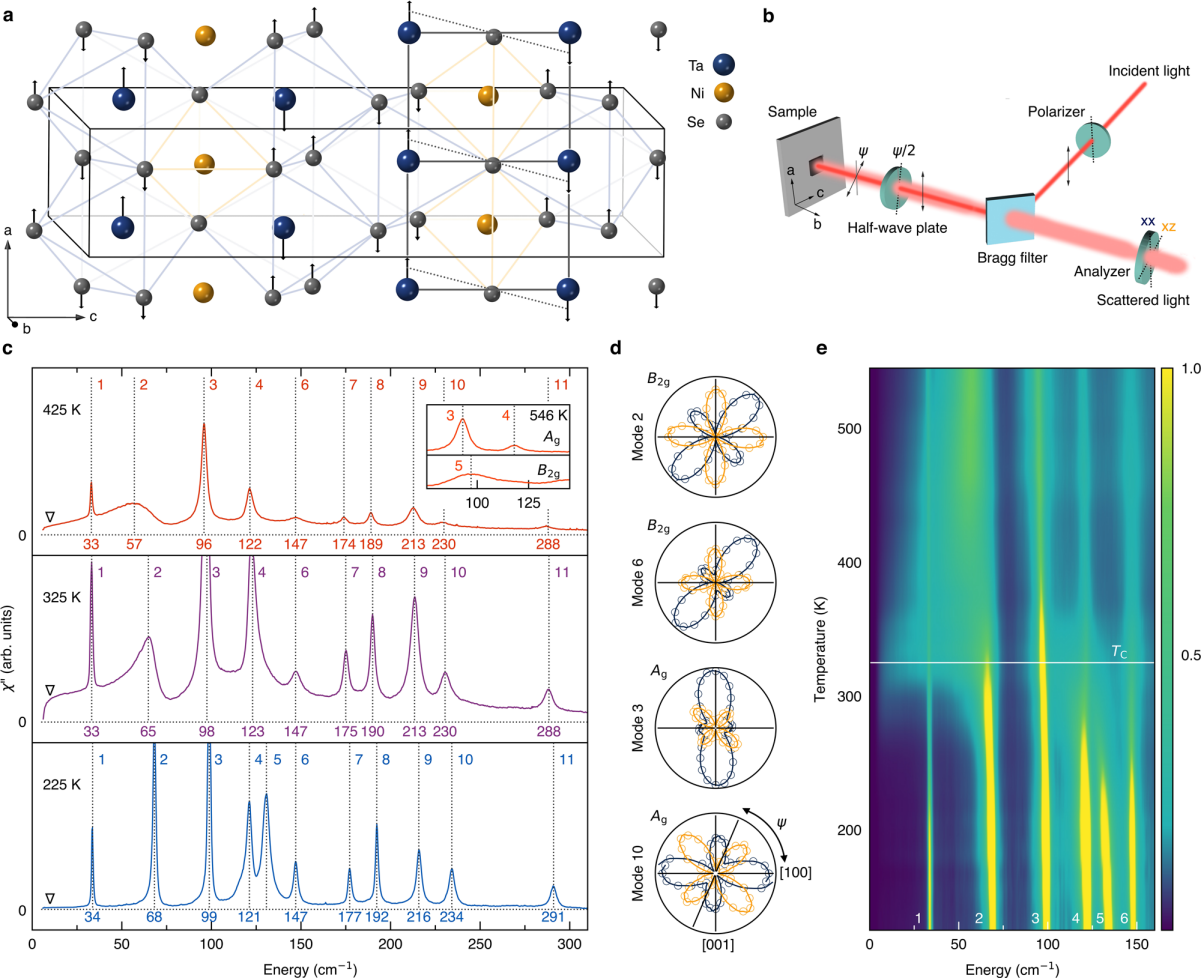


which replaces the previous incorrect version:
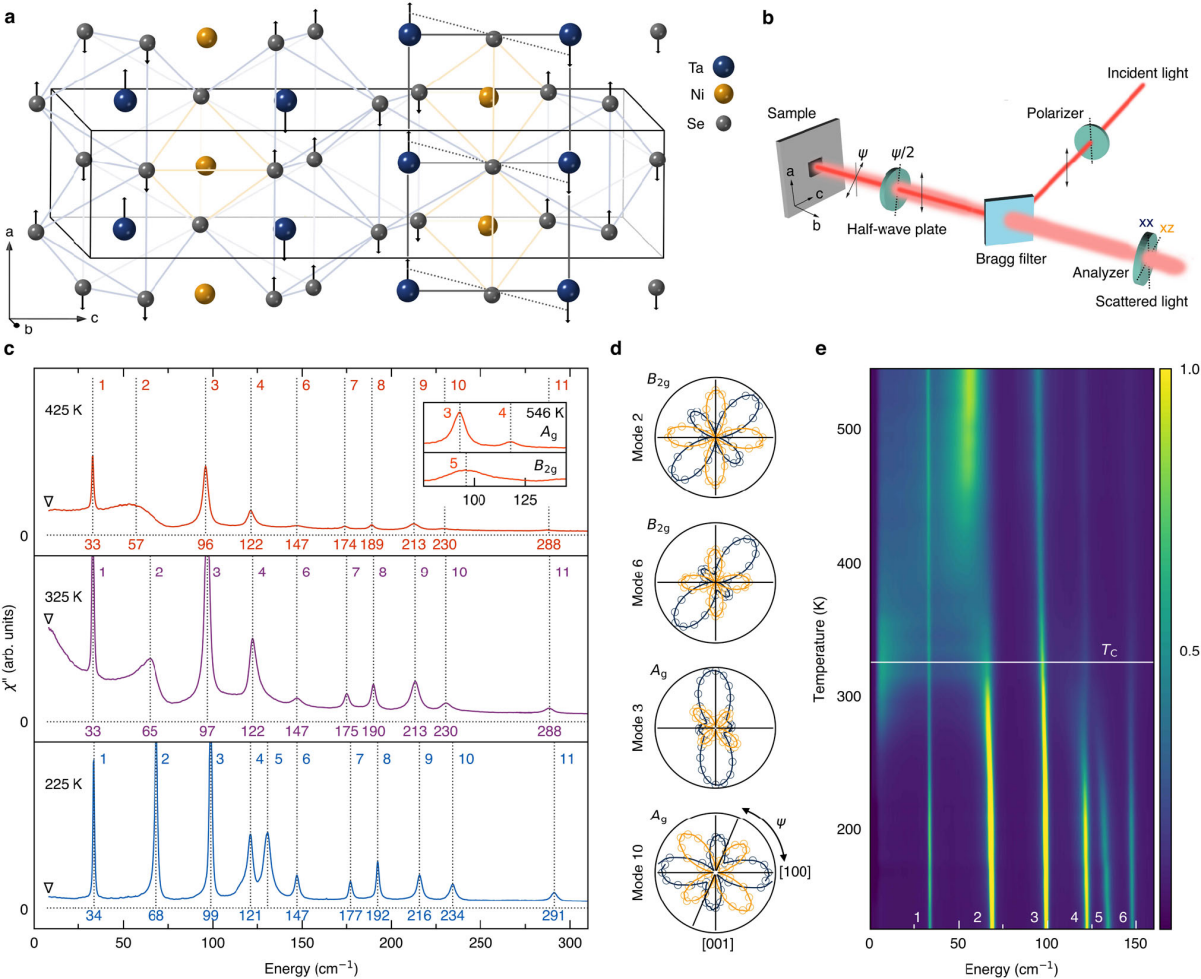


The original version of this Article contained an error in Fig. 2, in which the data was not corrected for the Bose factor.

The correct version is:
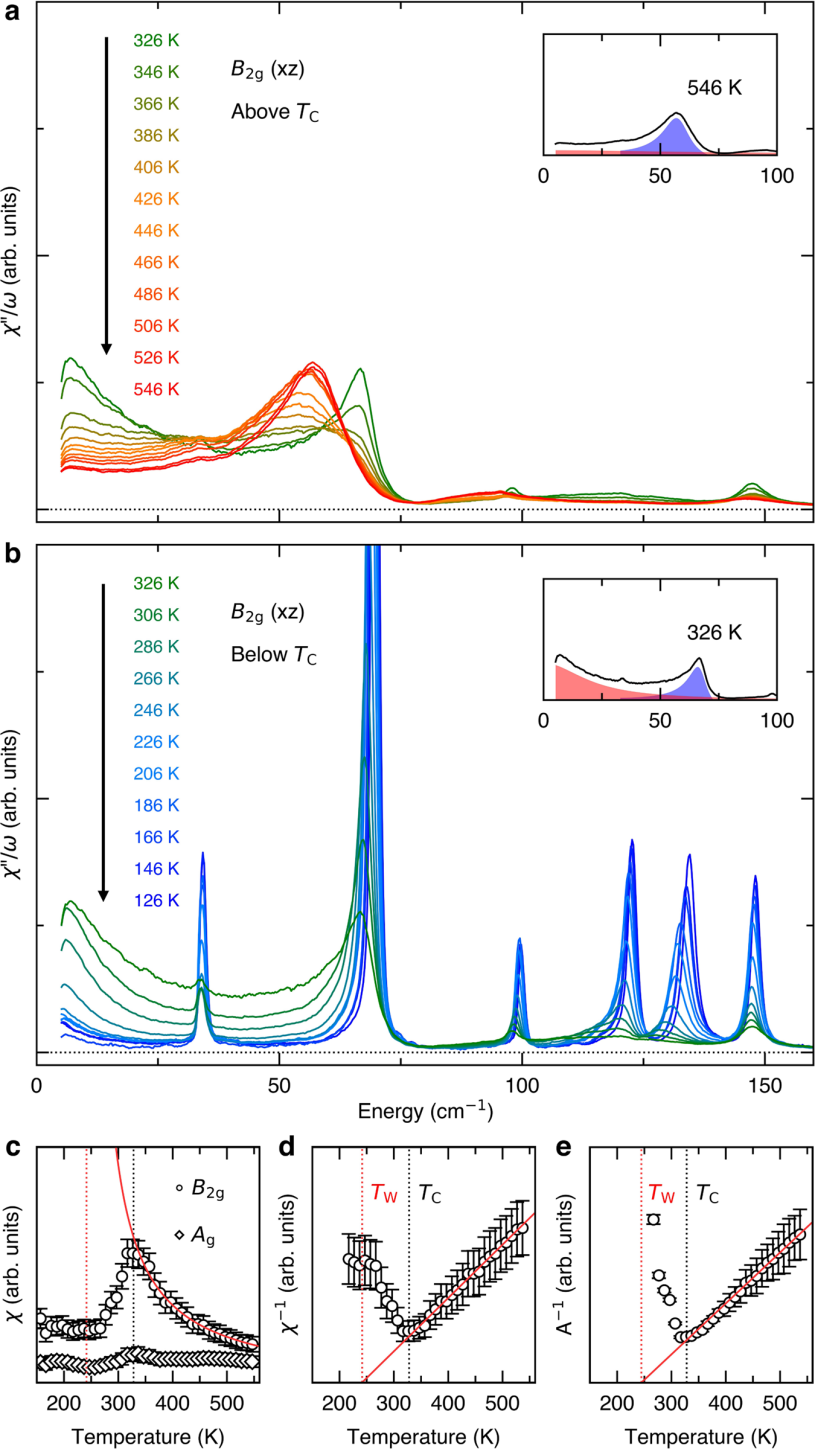


which replaces the previous incorrect version:
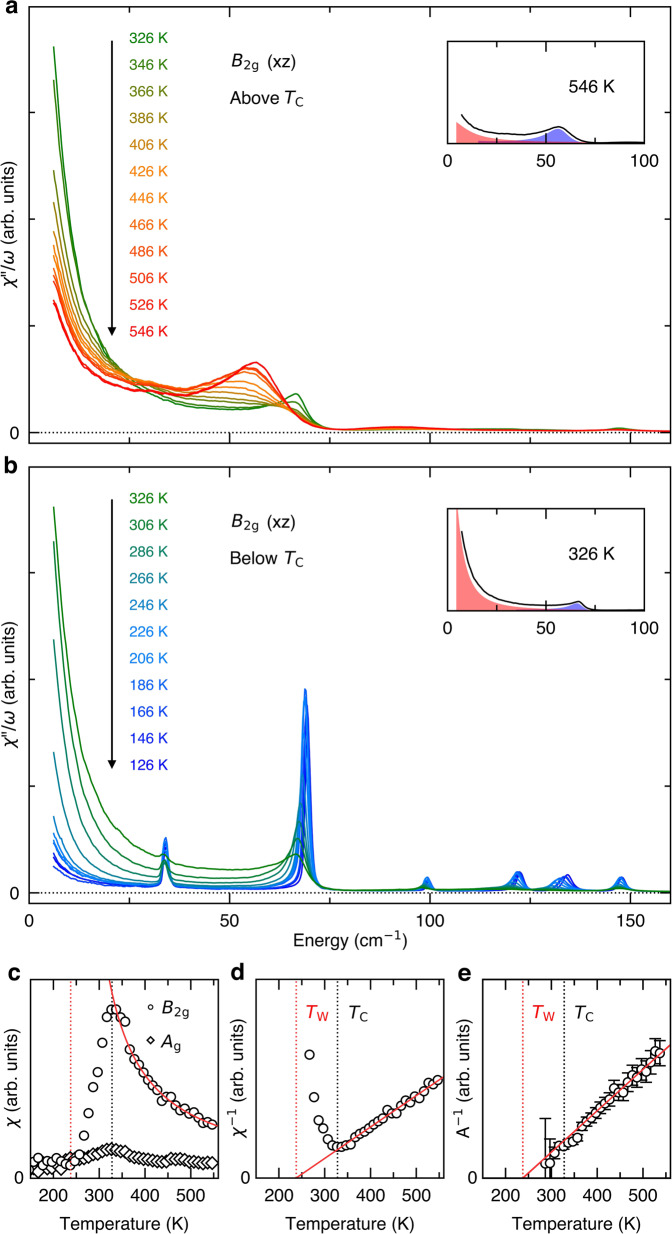


The original version of this Article contained an error in Fig. 3, in which the data was not corrected for the Bose factor.

The correct version is:
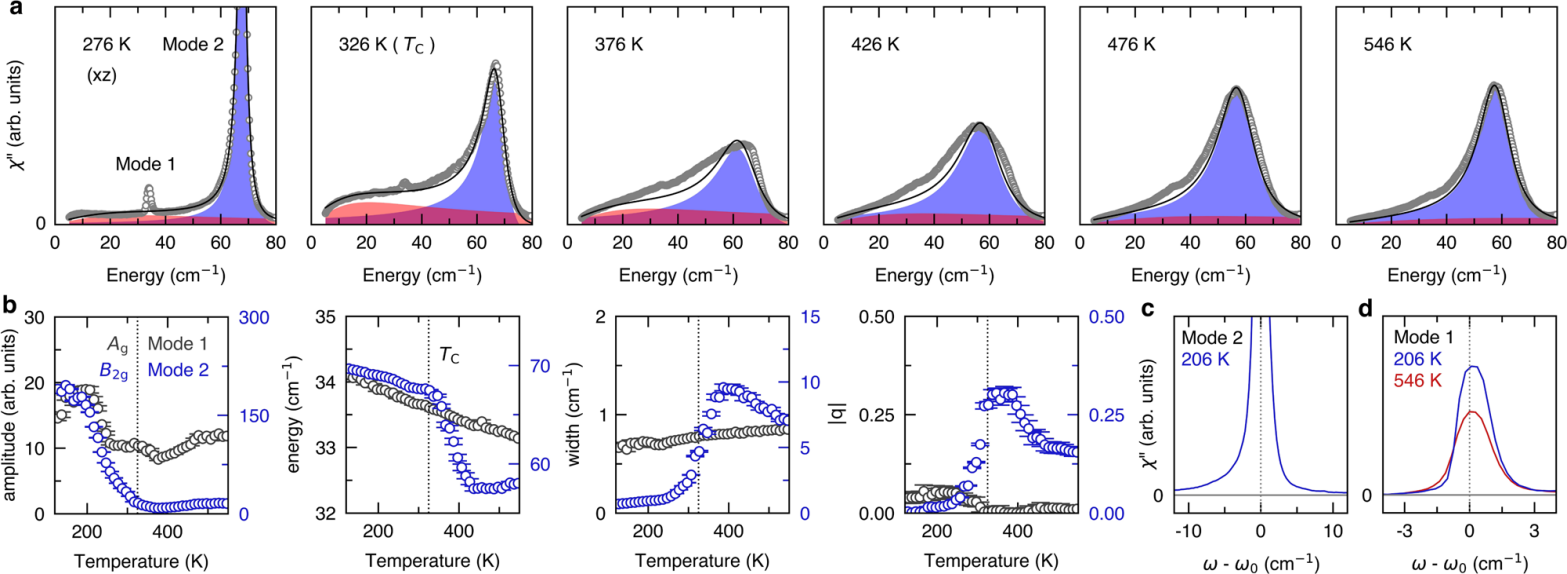


which replaces the previous incorrect version:
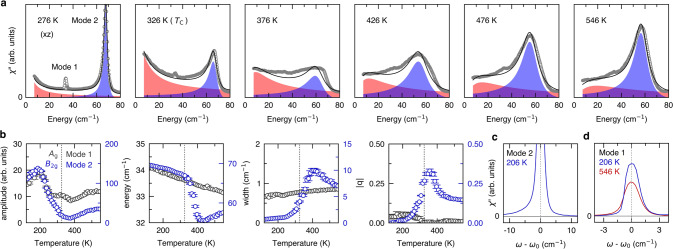


The original version of this Article contained an error in Fig. 4, in which the data was not corrected for the Bose factor.

The correct version is:
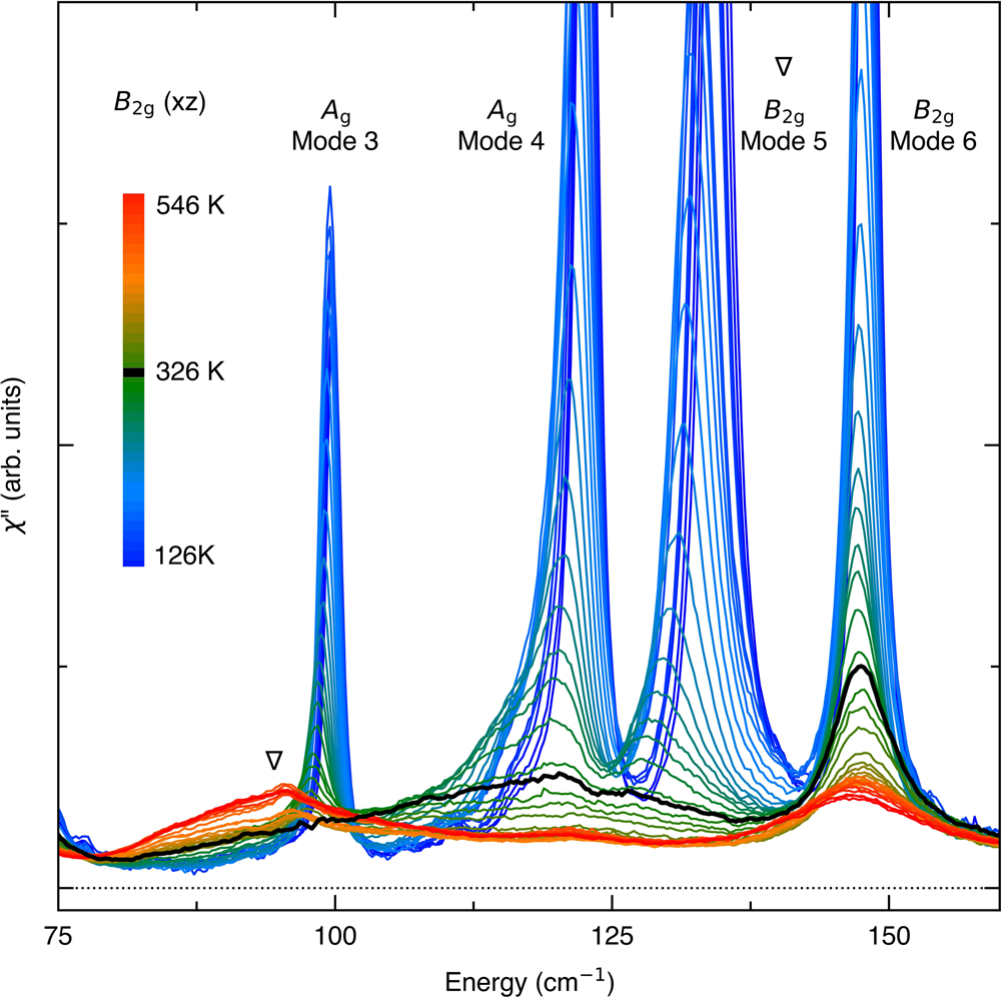


which replaces the previous incorrect version:
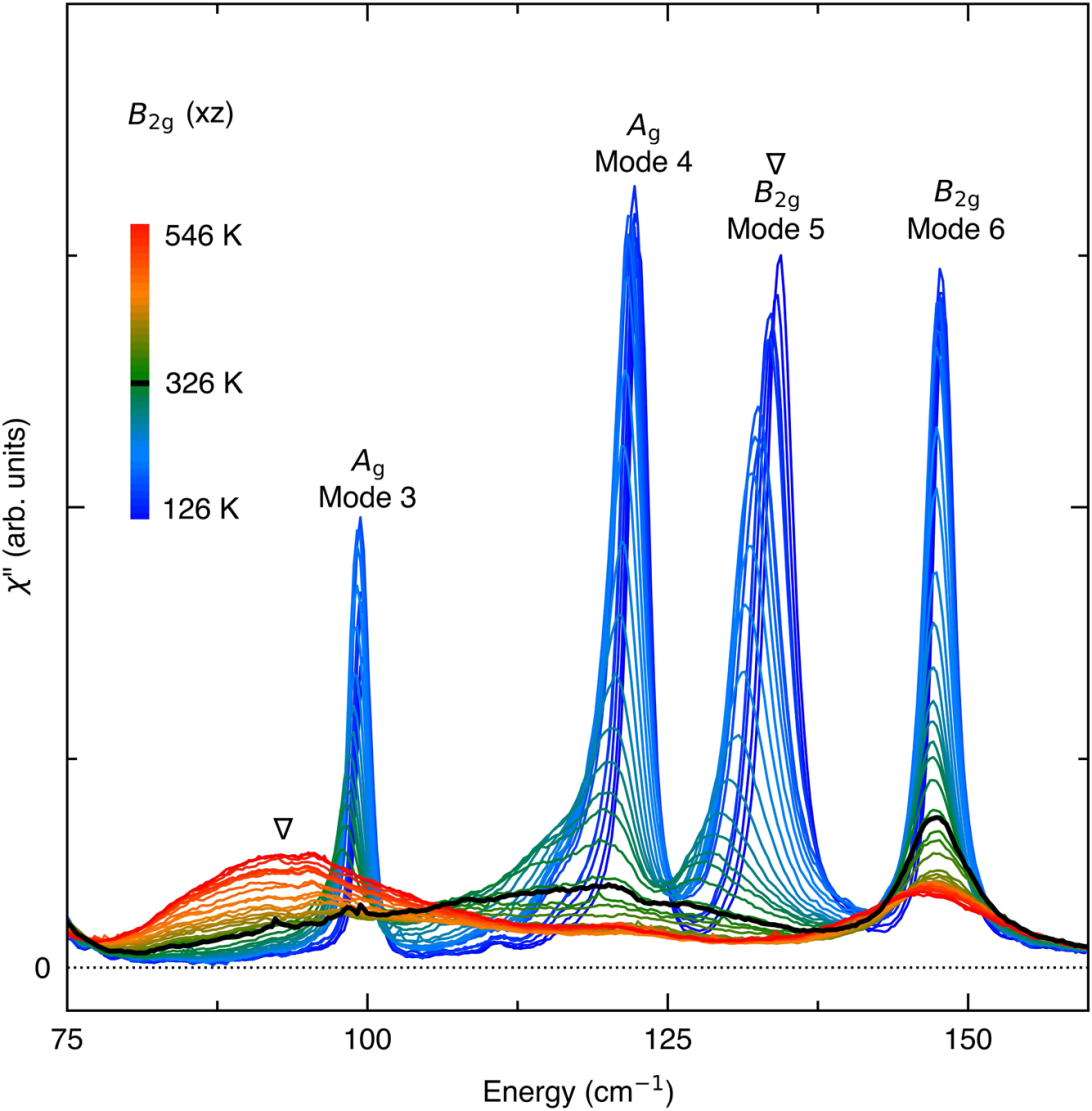


The original version of this Article contained an error in the fourth sentence of the Abstract, which incorrectly read “Critical fluctuations of the excitonic order parameter give rise to quasi-elastic scattering of B2g symmetry, whose intensity grows inversely with temperature toward the Weiss temperature of TW ≈ 237 K, which is arrested by a structural phase transition driven by an acoustic phonon of the same symmetry at TC = 325 K”. The correct version states “241” in place of “237”.

The first sentence of the sixth paragraph of the Results and discussion originally incorrectly read “The χB2g shows a Curie-Weiss behavior above TC with the Weiss temperature of 237 ± 5 K, extracted from linear extrapolation of the inverse susceptibility (Fig. 2c, d)”. The correct version states “241 ± 9” instead of “237 ± 5”.

The fourth sentence of the sixth paragraph of the Results and discussion originally incorrectly read “When extrapolated, they intercept the zero frequency at 238 K, giving a self-consistent quantification of the Weiss temperature”. The correct version states “244” instead of “238”.

This has been corrected in both the PDF and HTML versions of the Article.

