# Supplementary material for: Direct observation of excitonic instability in Ta2NiSe5
Source: Nat Commun. 2021 Mar 30;12:1969. doi: 10.1038/s41467-021-22133-z (PMC8010035; doi:10.1038/s41467-021-22133-z)
Supplement: Supplementary file 1 — Supplementary Information [file 41467_2021_22133_MOESM1_ESM.pdf]

Supplementary Information

Direct observation of excitonic instability in Ta_2NiSe_5

Kwangrae Kim,[1,2,*] Hoon Kim,[1,2,*] Jonghwan Kim,[2,3] Changil Kwon,[1,2] Jun Sung Kim,[1,2] and B. J. Kim[1,2,†]

[1]Department of Physics, Pohang University of Science and Technology, Pohang 37673, South Korea

[2]Center for Artificial Low Dimensional Electronic Systems, Institute for Basic Science (IBS), 77 Cheongam-Ro, Pohang 37673, South Korea

[3]Department of Materials Science and Engineering, Pohang University of Science and Technology, Pohang 37673, Republic of Korea

[*]These authors contributed equally to this work.

[†]Corresponding author (bjkim6@postech.ac.kr)

Supplementary Note 1: AZIMUTH ANGLE DEPENDENCE OF THE RAMAN-ACTIVE PHONON MODES

In the orthorhombic *Cmcm* phase, the Raman tensors have the following forms:

$$\hat{R}(A_{\mathrm{g}}) = \begin{pmatrix} c_1 & & \\ & c_2 & \\ & & c_3 \end{pmatrix}, \qquad \hat{R}(B_{2\mathrm{g}}) = \begin{pmatrix} & & c_4 \\ & & \\ c_4 & & \end{pmatrix}, \tag{1}$$

where c_1, c_2, c_3, and c_4 are non-zero elements. Further, c_2 is identically zero in our -y(xz)y configuration. For cross polarization at $\Psi = 0°$ (yellow patterns in Supplementary Fig. 1), A_{g} ($B_{2\mathrm{g}}$) Raman intensity vanishes (is maximized), because only off-diagonal components contribute to the intensity. Likewise, A_{g} Raman intensity is maximized at $\Psi = 45°$ while $B_{2\mathrm{g}}$ intensity is completely suppressed. This allows a clear separation of A_{g} and $B_{2\mathrm{g}}$ spectra as shown in the inset of Fig. 1c. Thus, mode 2, 5, 6 and QEP are unambiguously assigned as $B_{2\mathrm{g}}$ and the remaining eight modes as A_{g}. Although the azimuth profile of mode 5 at high temperature is not clearly of $B_{2\mathrm{g}}$ type due to its overlap with mode 3 and mode 4 of A_g symmetry, mode 5 is clearly identified as a well-defined peak in the $B_{2\mathrm{g}}$ spectrum shown in the inset of Fig. 1c.

Below T_{C}, A_g and B_{2g} are no longer distinct and the Raman tensor acquires the form:

$$\hat{R}(A_{\mathrm{g}}) = \begin{pmatrix} c_1 & & c_4 \\ & c_2 & \\ c_4 & & c_3 \end{pmatrix}. \tag{2}$$

The mixing between A_{g} and $B_{2\mathrm{g}}$ modes leads to intensity extrema deviating from the high-symmetry crystallographic directions (see, e.g., mode 1, 4, 7, and 9 in Supplementary Fig. 1). In Supplementary Table 1, we summarize the fitting results of the polarization dependence of all the modes, using Supplementary Eq. (1) and (2) at 200 K and 400 K, respectively.

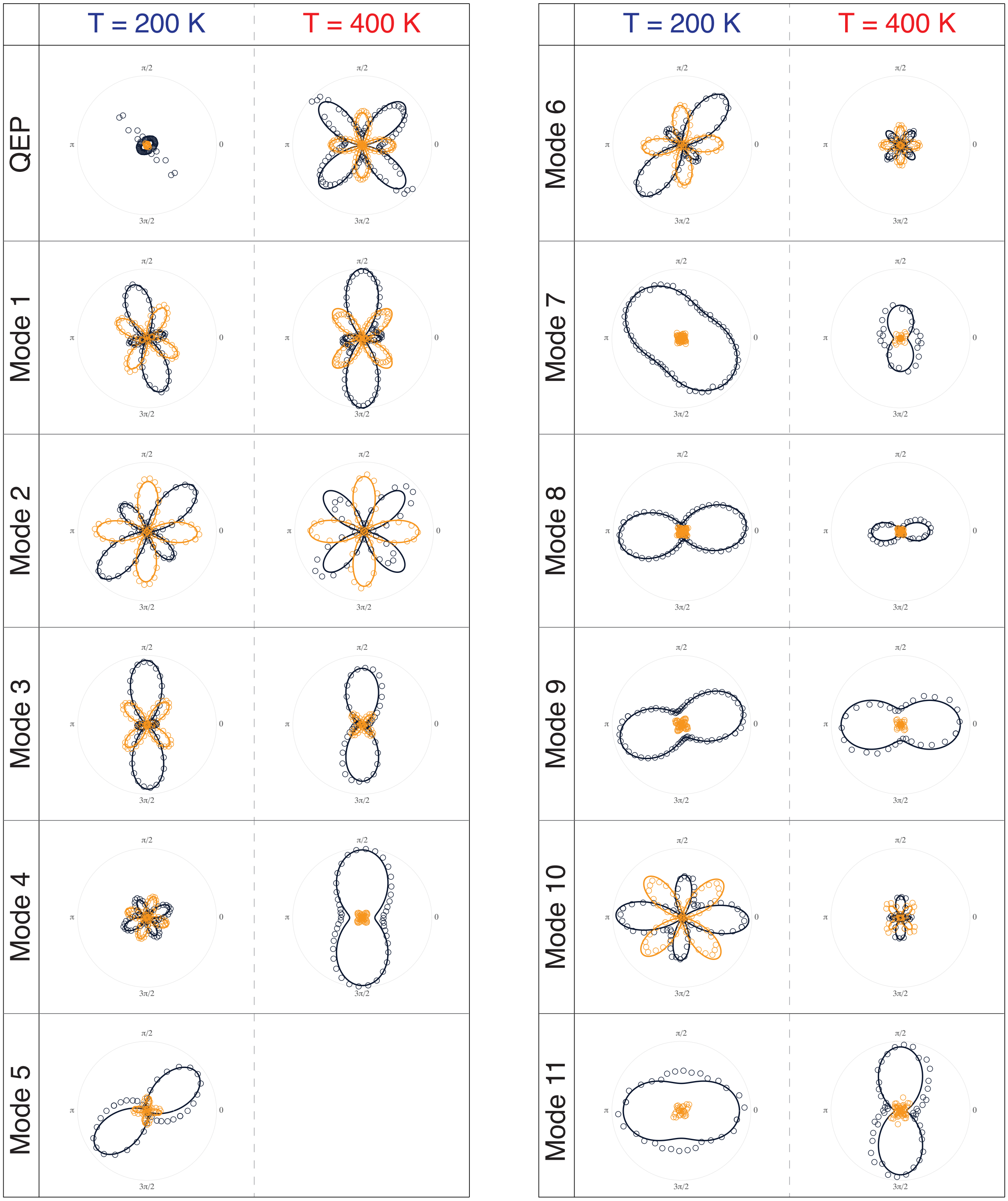

Supplementary Figure 1 | Azimuth-angle dependence of the the quasi-elastic peak and the phonon modes. Polar plot of Raman intensity as a function of the angle between the incident polarization and the a-axis below and above the T_C. Navy and yellow colors represent the parallel- and crosse-polarization configurations, respectively. Solid lines are fitting results shown in Supplementary Table 1.

Mode	IR	Orthorhombic ($Cmcm$) at 400 K			Monoclinic ($C2/c$) at 200 K		
		c_1	c_3	c_4	c_1	c_3	c_4
QEP	B_{2g}			1			
1	A_g	0.34	-0.66		0.27	-0.51	0.22
2	B_{2g}			1	0.15	0.07	0.78
3	A_g	-0.02	0.98		0.26	-0.71	0.02
4	A_g	0.30	0.70		-0.34	0.28	-0.39
5	B_{2g}				0.44	0.26	0.31
6	B_{2g}			1	0.10	0.26	0.64
7	A_g	0.32	0.68		0.48	0.45	-0.07
8	A_g	0.87	0.13		0.70	0.25	0.05
9	A_g	0.66	0.34		0.60	0.31	0.09
10	A_g	-0.41	0.59		-0.53	0.43	0.04
11	A_g	0.24	0.76		0.59	0.41	0

Supplementary Table 1 | Raman tensor elements. Fitting of the polarization dependence of the Raman modes, using the tensors given in Supplementary Eq. (1) and (2). The results are overlaid with the data points in Supplementary Fig. 1. The coefficients are normalized in such a way that $\|c_1\| + \|c_3\| + \|c_4\| = 1$.

Supplementary Note 2: FITTING RESULTS USING TWO DIFFERENT MODELS

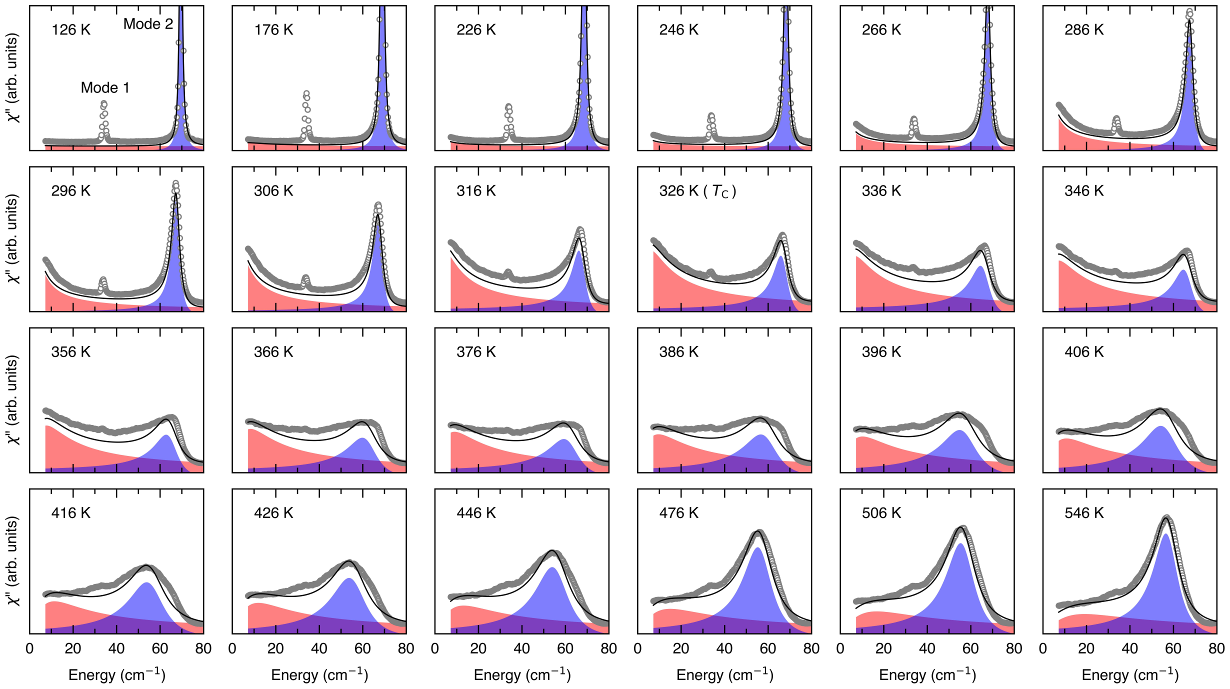

Supplementary Figure 2 | Fitting result using the Fano lineshape (blue) and the damped harmonic oscillator lineshape (red). All measured spectra at temperatures between 280 K and 420 K are shown.

In the main text, we analyze the spectra below 100 cm^{-1} using an independent electron-phonon model (Model 1) where we fit the QEP using damped harmonic oscillator lineshape and and mode 2 with Fano lineshape, expressed in Supplementary Eq. (3) and (4), respectively. The damped harmonic oscillator (or the Drude-Lorentz) is a typical model for electronically scattered Raman signals, and the Fano lineshape is generally used for phonon modes that couple to a continuum, where q determines their asymmetry, and thus their coupling strengths to the continuum. The lineshapes are expressed as

$$\chi''_{\text{electron}}(\omega) = \frac{A_{\text{el}}\Gamma_{\text{el}}\omega}{\Gamma_{\text{el}}^2 + \omega^2}, \tag{3}$$

$$\chi''_{\text{phonon}}(\omega) = \frac{A_{\text{ph}}(1+q\epsilon)^2}{(1+\epsilon)^2},\ \epsilon = \frac{\omega - \omega_{\text{ph}}}{\Gamma_{\text{ph}}}, \tag{4}$$

where χ''_{electron} and χ''_{phonon} is bose-corrected Raman intensity of QEP and optical phonon mode 2. A_{el}, Γ_{el}, A_{ph}, ω_{ph}, Γ_{ph}, and q indicate the amplitude, width of QEP, amplitude,

energy, width of mode 2, and the asymmetry of mode 2 respectively.

All measured spectra are fitted with the six adjustable parameters, and the result is shown in Supplementary Fig. 2. As shown in Fig. 2 and Supplementary Fig. 3, the extracted electronic susceptibility follows Curie-Weiss behavior above T_C with the Weiss temperature around 237 K. The inverse of QEP amplitude from fit shows a linear behavior above T_C, intercepting the zero at 238 K when extrapolated. In addition to parameters in Fig. 2, the width of QEP also increases linearly above T_C with an intercept at 239 K (Supplementary Fig. 3).

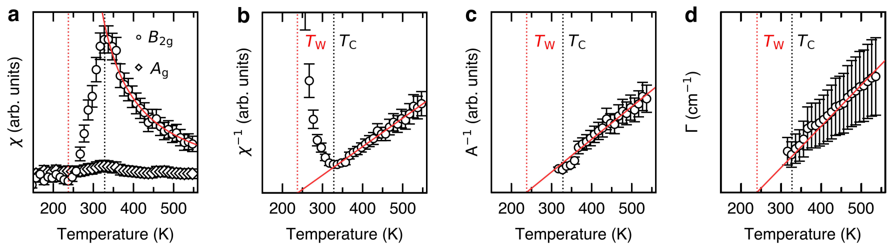

Supplementary Figure 3 | Fitting parameters for QEP. **a**, Electronic susceptibility in A_g (diamond) and B_{2g} (circle) channel. **b**, the inverse of B_{2g} electronic susceptibility. **c**, the inverse of QEP amplitude. **d**, the width of QEP. Error bars indicate the standard deviation from the fitting procedure.

In the below, we use a different model (Model 2), which takes into account the coupling between the QEP and mode 2, to fit the same spectra [1, 2]. In this model, the bare electron and the phonon Green functions, expressed as (with ω_{ph} and Γ_{ph} denoting energy and width of the phonon mode 2, respectively, and Γ_{el} the width of the QEP)

$$G_{\text{electron}}(\omega) = \frac{1}{\Gamma_{\text{el}} - i\omega}, \tag{5}$$

$$G_{\text{phonon}}(\omega) = -\frac{1}{\omega - \omega_{\text{ph}} + i\Gamma_{\text{ph}}} + \frac{1}{\omega + \omega_{\text{ph}} + i\Gamma_{\text{ph}}}, \tag{6}$$

are coupled by v to give the total Green function

$$G(\omega) = \begin{bmatrix} G^{-1}_{\text{electron}}(\omega) & v \\ v & G^{-1}_{\text{phonon}}(\omega) \end{bmatrix}^{-1}, \tag{7}$$

which is related to the Raman susceptibility via

$$\chi''(\omega) = \text{Im}\, TGT, \tag{8}$$

where $T = [t_{\mathrm{el}}\ t_{\mathrm{ph}}]$ is the amplitude for Raman light scattering process.

The fit result using this model is compared to the previous one (Model 1) in Supplementary Fig. 4. The amplitude, energy, and width of mode 2 are common for the two models, and thus can be directly compared to each other. We find an excellent agreement between the two sets of parameters. Although the q-asymmetry parameter in Model 1 and the coupling constant v in Model 2 have different physical meanings, they have similar temperature dependencies, reflecting the phonon anomaly that becomes pronounced around 400 K upon cooling.

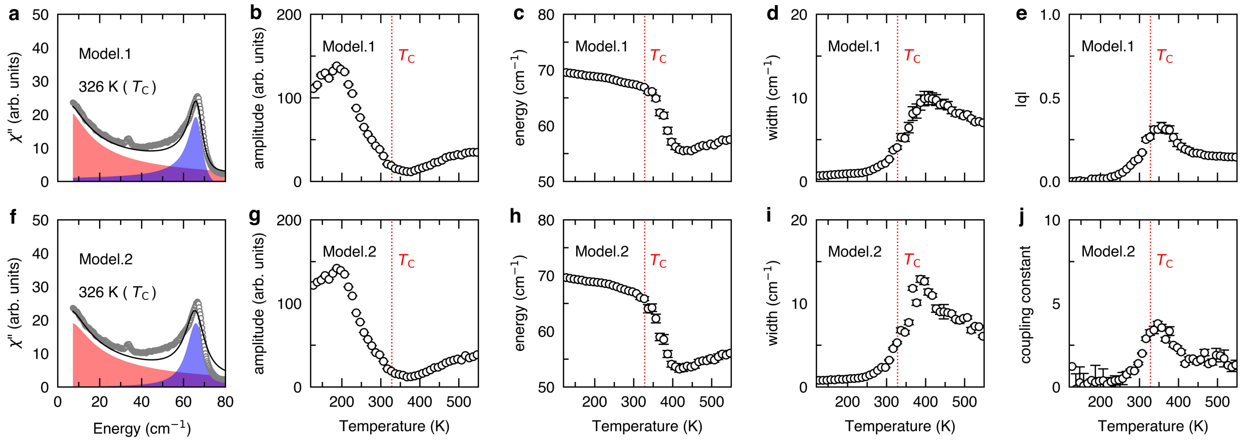

Supplementary Figure 4 | Fitting results for mode 2 with different fitting methods. **a**, QEP (red) fitted with the damped harmonic oscillator lineshape, and mode 2 (blue) with the Fano lineshape, respectively. **b-e**, amplitude, energy, width, and asymmetry of mode 2 from fitting method 1. **f**, QEP and mode 2 fitted with fitting method 2. Bare electronic feature (red) and mode 2 (blue) without electron-phonon coupling are shown. **g-j**, amplitude, energy, width, and asymmetry of mode 2 from fitting method 2.

Supplementary Note 3: IDENTIFICATION OF THE UNSTABLE OPTICAL MODE IN THE DENSITY FUNCTINOAL CALCULATION

Mode	IR	Orthorhombic ($Cmcm$)		Monoclinic ($C2/c$)	
		Exp. at 546 K (cm^{-1})	Calc. (cm^{-1})	Exp. at 100 K (cm^{-1})	Calc. (cm^{-1})
1	A_g	33	32.272	34	32.448
2	B_{2g}	57	74.755	69	59.704
3	A_g	95	94.880	99	95.020
4	A_g	120	120.515	122	120.569
5	B_{2g}	94		134	
6	B_{2g}	146	149.189	148	145.162
7	A_g	174	169.927	178	170.196
8	A_g	189	183.266	194	184.076
9	A_g	213	200.463	217	203.635
10	A_g	228	220.940	236	222.760
11	A_g	287	280.743	293	281.137

Supplementary Table 2 | Raman active phonon modes. Comparison between the calculated [3] and the measured mode energies. The IRs listed are based on the high-temperature phase. All of the phonon modes reduce to A_g IR in the monoclinic phase.

We identify the unstable optical mode in the DFT calculation to be the mode 5 in our data by comparing the measured phonon energies to the ones from the DFT calculation [3]. Their energies are in overall good agreement with each other except for the mode 5, which therefore is the one that has a negative energy in the DFT calculation. As shown in Fig. 4 and Supplementary Fig. 5, this mode does not soften to zero frequency, but hardens as the acoustic mode freezes.

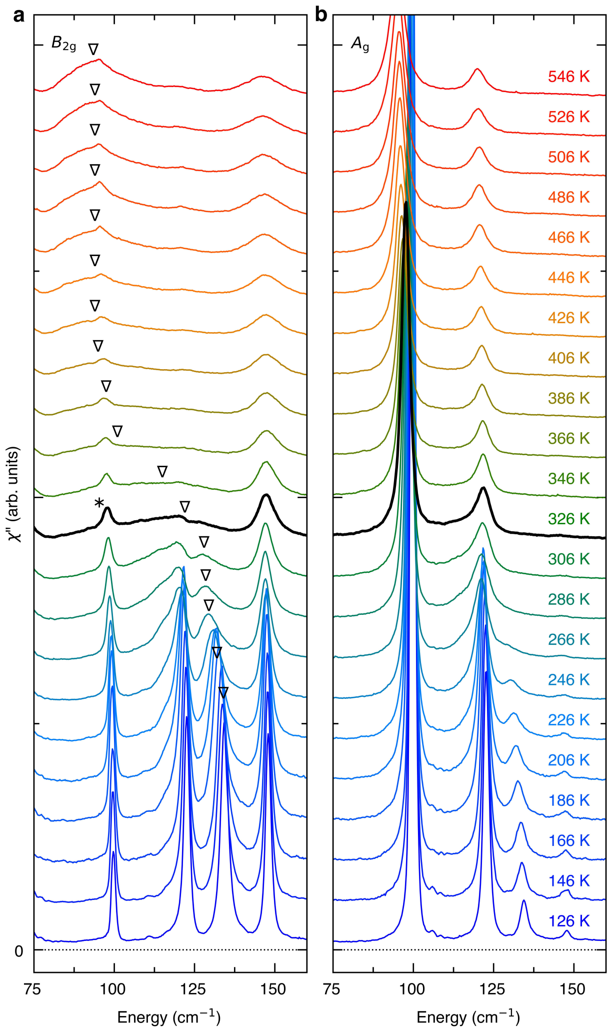

Supplementary Figure 5 | Vertically stacked Raman spectra. a, B_{2g} Raman spectra measured in -y(xz)y. **b**, A_g in -y(xx)y. Black solid line indicates T_C, and the leakage of A_g mode 3 is marked by an asterisk. Mode 5 (inverted triangle) is heavily damped at T_C, but never softens to zero energy.

[1] Klein, M. V. *"Electronic Raman Scattering" in Light Scattering in Solids I* **8**, 147-202 (Springer-Verlag Berlin, 1983)

[2] Volkov, P. A. et al. Critical charge fluctuations and quantum coherent state in excitonic insulator Ta_2NiSe_5. Preprint at http://arxiv.org/abs/2007.07344 (2020).

[3] Subedi, A. Orthorhombic-to-monoclinic transition in Ta_2NiSe_5 due to a zon-center optical phonon instability. *Phys. Rev. Mater.* **4**, 083601 (2020).
